# The influence of body mass index and outdoor temperature on the autonomic response to eating in healthy young Japanese women

**DOI:** 10.1186/2193-1801-3-142

**Published:** 2014-03-14

**Authors:** Masahiro Okada, Masayuki Kakehashi

**Affiliations:** Department of Food and Dietetics, Hiroshima Bunka Gakuen Two-Year College, 3-5-1 Nagatsukanishi, Asaminami-ku, Hiroshima, 731-0136 Japan; Graduate School of Biomedical & Health Sciences, Hiroshima University, Hiroshima, 734-8553 Japan

**Keywords:** Heart rate variability, Autonomic nervous system, Eating, Outdoor temperature, Body mass index

## Abstract

**Purpose:**

The influences of body weight and air temperature on the autonomic response to food intake have not been clarified. We measured heart rate variability before and after lunch, as well as the effects of outdoor temperature and increased body mass index (BMI), in healthy young Japanese women.

**Methods:**

We studied 55 healthy young female university students. Heart rate variability was measured before lunch, immediately after lunch, 30 min after lunch, and 1 h after lunch to determine any correlations between heart rate variability, outdoor temperature, and BMI. In addition, multiple regression analysis was performed to elucidate the relationship between heart rate variability and outdoor temperature before and after lunch. A simple slope test was conducted to show the relationship between the low-to-high frequency ratio (1 h after lunch) and outdoor temperature.

**Results:**

Subjects were divided into a low BMI group (range: 16.6–20.3) and a high BMI group (range: 20.4–32.9). The very low frequency component of heart rate variability, an index of thermoregulatory vasomotor control exerted by the sympathetic nervous system, was significantly diminished after lunch in the high BMI group (*P* < 0.01). A significant decrease in the low-to-high frequency (LF/HF) ratio, which represents the balance between the parasympathetic and sympathetic nervous systems, was evident in the low BMI group after lunch, indicating parasympathetic system dominance (*P* = 0.001). In addition, a significant association was found between the LF/HF ratio and outdoor temperature after lunch with a lower BMI (*P* = 0.002), but this association disappeared with higher BMIs.

**Conclusion:**

Autonomic responses to eating showed clear differences according to BMI, indicating that the sensitivity of the autonomic nervous system may change with increases in BMI.

## Background

The human body has a number of regulatory processes that maintain its internal conditions. Heart rate, body temperature, and respiration are examples of homeostatic responses that can be altered in response to internal changes. Although there have been numerous studies on autonomic responses to various influences, the effect of body weight and air temperature on the autonomic response to food intake have not been clarified. Furthermore, many of the previous studies have involved subjects with serious health issues (e.g., obesity, diabetes, anorexia), thus very few have been conducted on healthy subjects (i.e., with no major illness and within the recommended body mass index [BMI]).

There has been a great deal of interest in the relationships between the autonomic nervous system (ANS) and metabolic syndrome, including obesity. Furthermore, several components of heart rate variability appear to be influenced by obesity (Piccirillo et al. 1996; Matsumoto et al. 1999,2001; Tentolouris et al. 2003; Fujibayashi et al. 2009; Wijngaarden et al. 2012), diabetes mellitus (Kataoka et al. 2004; Franklin et al. 2008; Sjoberg et al. 2011), and hypertension (Boer-Martins et al. 2011; Pal et al. 2012). Heart rate variability has been used to evaluate the homeostatic responses to food intake (Lu et al. 1999; Dionne et al. 2002; Harthoorn and Dransfield 2008), environmental factors (e.g., weather, temperature: Bruce-Low et al. 2006; Liu et al. 2008), and body composition (Molfino et al. 2009; Sztajzel et al. 2009; Soares-Miranda et al. 2011; Andrew et al. 2013; Yi et al. 2013).

In 1991, Bray proposed the MONA LISA (Most Obesities kNown Are Low In Sympathetic Activity) hypothesis (Bray 1991), which has been studied by a variety of means, including heart rate variability. The very low frequency (VLF) and low frequency (LF) components, which serve as indices of sympathetic dominance, appear to be diminished in obesity (Piccirillo et al. 1996; Matsumoto et al. 1999,2001; Tentolouris et al. 2003). After food intake and cold exposure, the VLF component in obese women has been found to be lower than that of controls (Matsumoto et al. 1999,2001). When obese subjects reduced their body weight, the VLF (which may be affected by blood pressure and microvascular variability) and LF components tended to increase (Fujibayashi et al. 2009; Sjoberg et al. 2011; Wijngaarden et al. 2012).

The relationships between adiposity and heart rate variability have been investigated using several indices of body composition (Molfino et al. 2009; Sztajzel et al. 2009; Soares-Miranda et al. 2011; Andrew et al. 2013; Yi et al. 2013) including BMI, which has been validated for use in Asians (Stevens and Nowicki 2003; Takimoto et al. 2004). The MONA LISA hypothesis has been proven (Piccirillo et al. 1996; Matsumoto et al. 1999,2001; Tentolouris et al. 2003; Fujibayashi et al. 2009; Wijngaarden et al. 2012), and correlations between heart rate variability and BMI have been found in patients with type II diabetes mellitus and hypertension (Kataoka et al. 2004; Boer-Martins et al. 2011; Sjoberg et al. 2011; Pal et al. 2012) and heart rate variability and metabolic syndrome in women (Matsumoto et al. 1999; Matsumoto et al. 2001; Tentolouris et al. 2003; Franklin et al. 2008; Fujibayashi et al. 2009; Sztajzel et al. 2009; Soares-Miranda et al. 2011). However, detailed relationships between heart rate variability and BMI remain unclear in healthy humans (Molfino et al. 2009; Yi et al. 2013).

Outdoor temperature has been shown to influence human metabolism, even if the inside temperature is moderate. Kashiwazaki et al. (1990) found that a cooler outdoor temperature affects postprandial resting metabolic rate and Nedergaard et al. (2007) showed that active brown adipose tissue, related to thermogenesis, may be influenced by outdoor temperature. However, the effect of eating, outdoor temperature and BMI on heart rate variability have not been investigated in detail. A better understanding of the relationships between these factors could help to further determine the association between body weight and ANS responses. We hypothesized that even in healthy humans, the effects of food intake and outdoor temperature on ANS responses to eating might vary with differences in body weight. We measured heart rate variability before and after lunch and assessed the effects of outdoor temperature and increased BMI on the autonomic response to eating in healthy women.

## Results

Table 1 shows the demographic characteristics of the subjects recorded during the study periods (n = 55). The BMI distribution of the 55 subjects was as follows: 8 subjects <18.5 kg/m^2^, 15%; 42 subjects 18.5–25.0 kg/m^2^, 76%; and 5 subjects >25.0 kg/m^2^, 9%. The median BMI was 20.3 kg/m^2^ (range: 16.6–32.9). Other than BMI, weight and body fat proportion, there were no significant differences in other participant characteristics or outdoor environmental factors between the groups. See Table 1 for the recorded environmental factors.

**Table 1:** **Characteristics of the study population, outdoor environmental factors, and differences between BMI groups**

Groups	Range		High BMI group		Low BMI group		Difference	
Characteristics	Mean ± SE	Range	Mean ± SE	Range	Mean ± SE	Range	***P*** value	95% CI
Participants	n = 55		n = 26		n = 29			
Age (years)	20.4 ± 0.4	18–29	20.5 ± 0.6	18–29	20.2 ± 0.4	18–28	0.676	-0.12–1.72
Height (m)	1.58 ± 0.01	1.46–1.72	1.57 ± 0.01	1.47–1.66	1.58 ± 0.01	1.46–1.72	0.217	-0.05–0.01
Weight (kg)	52.2 ± 1.1	41.7–72.6	57.5 ± 1.3	46.5–72.6	47.5 ± 0.7	41.7–55.7	<0.001	6.97–13.06
BMI (kg/m^2^)	20.9 ± 0.4	16.6–32.9	23.3 ± 0.6	20.4–32.9	18.8 ± 0.2	16.6–20.3	<0.001	3.30–5.79
Body fat proportion (%)	28.8 ± 0.7	17.4–40.9	32.3 ± 0.8	26.3–40.9	25.7 ± 0.7	17.4–33.0	<0.001	4.51–8.77
Outdoor environmental factors								
Outdoor temperature (°C)	18.1 ± 1.2	1.3–31.6	17.3 ± 1.8	2.8–30.9	18.9 ± 1.7	1.3–31.6	0.496	-6.62–3.25
Atmospheric pressure (hPa)	1007.4 ± 0.9	994.7–1022.4	1008.3 ± 1.3	998.2–1021.1	1006.6 ± 1.1	994.7–1022.4	0.343	-1.81–5.10
Relative humidity (%)	67.0 ± 1.4	41.0–89.0	66.9 ± 2.4	41.0–89.0	67.1 ± 1.7	47.0–88.0	0.951	-5.66–5.64

SE = standard error.

BMI = body mass index.

*P* values represent the difference between the high and low BMI groups.

CI = confidence interval.

Analysis of the characteristics of the two groups revealed no significant differences with the exception of weight (*P* < 0.001, 95% CI 6.97–13.6), BMI (*P* < 0.001, 95% CI 3.30–5.79) and body fat percentage (*P* < 0.001, 95% CI 4.51–8.77). We also found no significant differences in the environmental factors experienced by the two groups.

**Figure 1:**
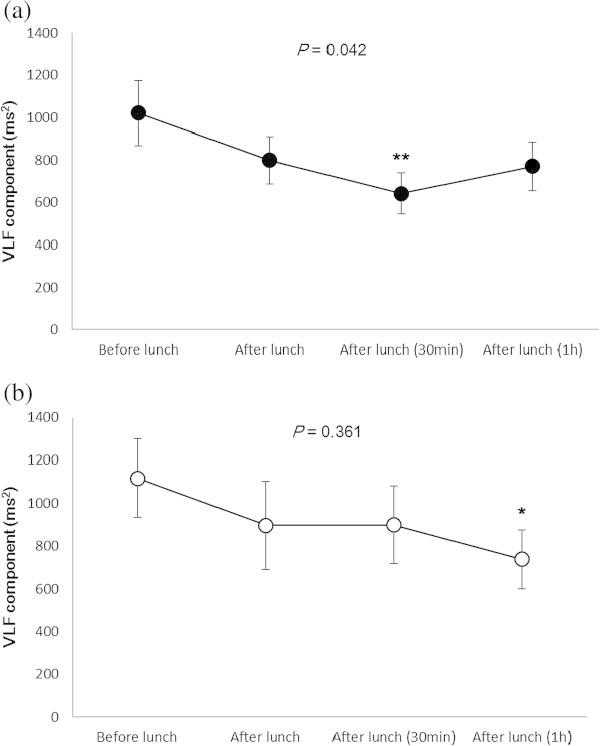
**Changes in the very low frequency component before and after lunch. (a)** High and **(b)** low BMI groups. Values are expressed as the mean ± standard error. *P* value was calculated using the Friedman test. The Wilcoxon signed-rank test was used to compare heart rate variability (before vs. after lunch, **P < 0.01, *P < 0.05).

**Figure 2:**
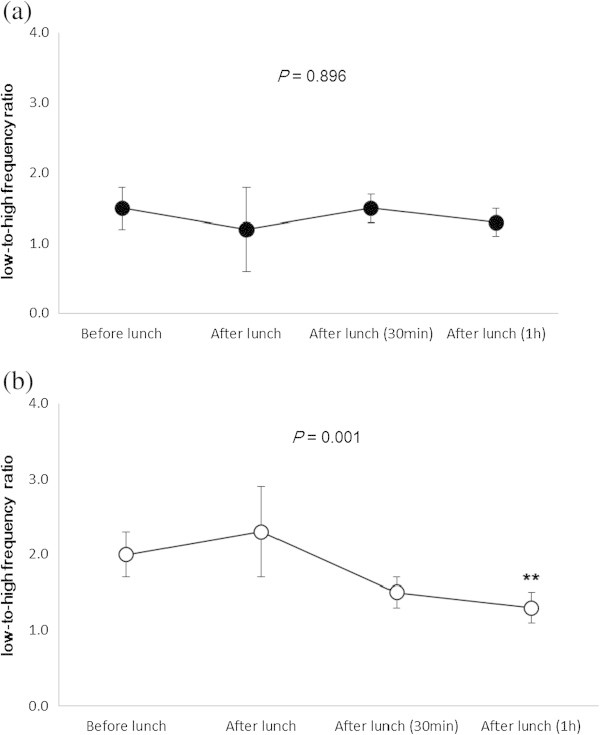
**Changes in the low-to-high frequency ratio before and after lunch. (a)** High and **(b)** low BMI groups. Values are expressed as the mean ± standard error. *P* value was calculated using the Friedman test. The Wilcoxon signed-rank test was used to compare heart rate variability (before vs. after lunch, **P < 0.01).

Table 2a shows heart rate and heart rate variability for all subjects before and after lunch. The heart rate was significantly higher after lunch (*P* < 0.01). The VLF component was significantly decreased 30 min (*P* = 0.015) and 1 h (*P* = 0.008) after lunch, and the LF/HF ratio significantly decreased 1 h after lunch (*P* = 0.005). In the high BMI group, we found that the VLF component of heart rate variability fell significantly after lunch (*P* = 0.042), particularly 30 min after eating (*P* < 0.01, Figure 1a); however, there was no significant change in the LF/HF ratio before and after lunch (Figure 2a). In the low BMI group, we found a less pronounced decrease in the VLF component 1 h after lunch (*P* < 0.05, Figure 1b), a significant change in the LF/HF ratio (*P* = 0.001) before and after lunch, and a significantly lower ratio 1 h after lunch (*P* < 0.01, Figure 2b). There were no significant differences in other heart rate variability components between groups. We found no correlations between other measured variables and BMI in all subjects, although the LF component was weakly correlated with BMI 1 h after lunch (*P* = 0.023, Table 2b).

**Table 2:** **Change in heart rate variability and its relationship with body mass index in all subjects (n = 55)**

(a) Variables	Before lunch	After lunch	After lunch (30 min)	After lunch (1 h)
Heart rate (beats/min)	73.4 ± 1.4	77.9 ± 1.5 (< 0.001)	78.3 ± 1.6 (< 0.001)	76.4 ± 1.6 (0.007)
Very low frequency (ms^2^)	1070.3 ± 120.2	849.2 ± 120.1 (0.129)	776.2 ± 107.0 (0.015)	752.2 ± 89.8 (0.008)
Low frequency (ms^2^)	772.6 ± 105.2	823.6 ± 107.3 (0.625)	624.5 ± 86.7 (0.162)	608.9 ± 60.5 (0.212)
High frequency (ms^2^)	643.1 ± 73.4	695.6 ± 88.6 (0.331)	611.7 ± 72.0 (0.688)	818.4 ± 110.7 (0.108)
Low-to-high frequency ratio	1.8 ± 0.2	1.8 ± 0.3 (0.703)	1.5 ± 0.2 (0.272)	1.3 ± 0.2 (0.005)
**(b) Variables**	**Before lunch**	**After lunch**	**After lunch (30 min)**	**After lunch (1 h)**
Heart rate (beats/min)	-0.224 (0.099)	-0.123 (0.372)	-0.218 (0.110)	-0.226 (0.098)
Very low frequency (ms^2^)	-0.073 (0.595)	0.159 (0.246)	0.006 (0.967)	0.116 (0.397)
Low frequency (ms^2^)	0.189 (0.168)	-0.094 (0.494)	0.097 (0.479)	0.306 (0.023)
High frequency (ms^2^)	0.201 (0.142)	0.089 (0.518)	0.175 (0.201)	0.143 (0.298)
Low-to-high frequency ratio	-0.037 (0.790)	-0.193 (0.157)	-0.085 (0.536)	0.103 (0.455)

(a) Difference in heart rate variability before and after lunch.

Mean ± standard error.

Parentheses are *p* values compared with values before lunch (Wilcoxon-signed rank test).

(b) Correlation between heart rate variability and BMI before and after lunch.

Spearman’s rank correlation coefficients are shown, *p* values in parentheses.

**Figure 3:**
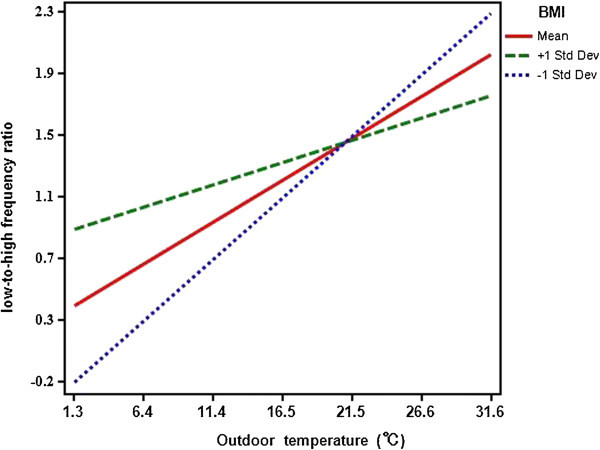
**Slopes showing the relationship between the low-to-high frequency ratio (1 h after lunch) and the outdoor temperature.** Mean (regression coefficients = 0.055, *P* values = 0.004), +1 standard deviation (regression coefficients = 0.029, *P* = 0.318), -1 standard deviation (regression coefficients = 0.081, *P* = 0.002).

Table 3 shows the relationships between heart rate, heart rate variability, and outdoor temperature before and after lunch in all subjects. The LF component was positively correlated with outdoor temperature 1 h after lunch (r = 0.302, *P* = 0.025), and the LF/HF ratio was positively correlated with outdoor temperature before (r = 0.303, *P* = 0.024) and 1 h after lunch (r = 0.398, *P* = 0.003). In the high BMI group, we found no correlations between any of the variables and outdoor temperature. In the low BMI group, outdoor temperature was positively correlated with the LF component 1 h after lunch (r = 0.466, *P* = 0.011), and the LF/HF ratio before lunch (r = 0.495, *P* = 0.006) and 1 h after lunch (r = 0.478, *P* = 0.009). The simple slope test in Figure 3 shows the relationship between the LF/HF ratio and outdoor temperature 1 h after lunch. A significant relationship was found between outdoor temperature and lower BMI (*P* = 0.002) but this disappeared as the BMI increased (*P* = 0.318).

**Table 3:** **Correlations between heart rate variability and outdoor temperature before and after lunch**

Variables	Before lunch	After lunch	After lunch (30 min)	After lunch (1 h)
**All subjects (n = 55)**				
Heart rate	0.064 (0.642)	0.047 (0.731)	-0.019 (0.889)	0.053 (0.701)
Very low frequency	0.125 (0.363)	-0.063 (0.647)	0.064 (0.641)	0.236 (0.082)
Low frequency	0.051 (0.710)	0.073 (0.594)	0.117 (0.393)	0.302 (0.025)
High frequency	-0.250 (0.066)	-0.134 (0.328)	-0.143 (0.298)	-0.162 (0.236)
Low-to-high frequency ratio	0.303 (0.024)	0.237 (0.081)	0.230 (0.090)	0.398 (0.003)
**High BMI group (n = 26)**	**Before**	**After**	**After (30 min)**	**After (1 h)**
Heart rate	0.131 (0.525)	0.103 (0.618)	-0.040 (0.847)	0.017 (0.819)
Very low frequency	0.189 (0.355)	-0.268 (0.186)	0.056 (0.784)	0.180 (0.379)
Low frequency	-0.116 (0.572)	0.001 (0.999)	0.247 (0.224)	0.162 (0.429)
High frequency	-0.112 (0.587)	-0.137 (0.504)	-0.133 (0.518)	-0.186 (0.362)
Low-to-high frequency ratio	0.019 (0.926)	0.201 (0.324)	0.381 (0.055)	0.324 (0.106)
**Low BMI group (n = 29)**	**Before**	**After**	**After (30 min)**	**After (1 h)**
Heart rate	-0.034 (0.861)	-0.040 (0.837)	-0.057 (0.768)	-0.013 (0.946)
Very low frequency	0.080 (0.679)	0.101 (0.602)	0.115 (0.553)	0.302 (0.111)
Low frequency	0.167 (0.385)	0.076 (0.696)	-0.004 (0.982)	0.466 (0.011)
High frequency	-0.269 (0.158)	-0.141 (0.466)	-0.135 (0.484)	-0.130 (0.501)
Low-to-high frequency ratio	0.495 (0.006)	0.292 (0.125)	0.121 (0.533)	0.478 (0.009)

BMI = body mass index.

Spearman’s rank correlation coefficients are shown; *P* values are in parentheses.

Table 4 shows the relationships between heart rate, heart rate variability before and after lunch, and outdoor temperature. Increased outdoor temperature was associated with an increased LF component 1 h after lunch (standard regression coefficient (β) = 0.370, *P* = 0.045). Decreased outdoor temperature was associated with an increased HF component before lunch (β = -0.461, *P* = 0.014), 30 min after lunch (β = -0.475, *P* = 0.013), and 1 h after lunch (β = -0.585, *P* = 0.002). Decreased outdoor temperature was associated with a decreased LF/HF ratio 1 h after lunch (β = -0.506, *P* = 0.004). We found no significant associations between any of the variables and outdoor temperature in the high BMI group; however, in the low BMI group, lower outdoor temperature was associated with an increased HF component before lunch (β = -0.540, *P* = 0.021) and a decreased LF/HF ratio 1 h after lunch (β = 0.610, *P* = 0.021).

**Table 4:** **Relationships between heart rate variability and outdoor temperature before and after lunch**

Variables	Before lunch	After lunch	After lunch (30 min)	After lunch (1 h)
**All subjects (n = 55)**				
Heart rate	-0.092 (0.592)	-0.117 (0.539)	-0.086 (0.645)	0.049 (0.793)
Very low frequency	-0.028 (0.881)	0.104 (0.586)	0.164 (0.402)	0.120 (0.517)
Low frequency	-0.282 (0.128)	-0.035 (0.860)	0.026 (0.894)	0.370 (0.045)
High frequency	-0.461 (0.014)	-0.364 (0.061)	-0.475 (0.013)	-0.585 (0.002)
Low-to-high frequency ratio	0.349 (0.071)	0.274 (0.168)	0.328 (0.089)	0.506 (0.004)
**High BMI group (n = 26)**	**Before**	**After**	**After (30 min)**	**After (1 h)**
Heart rate	-0.005 (0.987)	-0.090 (0.834)	-0.038 (0.924)	0.280 (0.473)
Very low frequency	0.427 (0.314)	-0.039 (0.920)	-0.055 (0.901)	0.012 (0.976)
Low frequency	-0.414 (0.277)	0.045 (0.920)	0.409 (0.271)	0.428 (0.281)
High frequency	-0.065 (0.877)	-0.077 (0.854)	-0.208 (0.623)	-0.464 (0.203)
Low-to-high frequency ratio	-0.405 (0.178)	-0.226 (0.603)	-0.452 (0.256)	-0.092 (0.756)
**Low BMI group (n = 29)**	**Before**	**After**	**After (30 min)**	**After (1 h)**
Heart rate	-0.252 (0.245)	-0.244 (0.335)	-0.166 (0.513)	-0.119 (0.632)
Very low frequency	-0.135 (0.557)	0.169 (0.534)	0.286 (0.283)	0.110 (0.663)
Low frequency	-0.228 (0.332)	-0.021 (0.938)	-0.020 (0.944)	0.366 (0.138)
High frequency	-0.540 (0.021)	-0.333 (0.200)	-0.382 (0.107)	-0.451 (0.060)
Low-to-high frequency ratio	0.531 (0.050)	0.224 (0.439)	0.302 (0.259)	0.610 (0.021)

BMI = body mass index.

Analysis was adjusted for room temperature, age, height, weight, BMI, body fat percentage, atmospheric pressure, and relative humidity.

Values are shown are the standard regression coefficients (β), with *p* values in parentheses.

## Discussion

We found that the heart rate tended to increase after lunch, a finding that is consistent with other studies (Lu et al. 1999; Harthoorn and Dransfield 2008). The VLF of heart rate variability, an indicator of sympathetic nervous system dominance, decreased from just after lunch to 1 h after lunch. In contrast, it was found that there was a tendency for the HF component, an indicator of parasympathetic dominance, to increase 1 h after lunch. The LF/HF ratio, an indicator of autonomic nervous balance, decreased 1 h after lunch. The relationships between the increased heart rate (indicating increased sympathetic tone) and decreased LF/HF ratio (indicating increased parasympathetic tone) after lunch may appear to be contradictory. However, Harthoorn and Dransfield (2008) also reported differences between the heart rate and the LF/HF ratio after a meal. While the heart rate was significantly increased after a meal, the LF/HF change was transient and not significant. We suggest that heart rate is strongly affected by the balance between sympathetic tone and vagal drive after lunch. We found that the peak heart rate occurred immediately or 30 min after lunch. From 30 minutes after lunch, we believe that the vagal drive became gradually predominant (decreasing the LF/HF ratio) and slowly inhibited sympathetic tone. Consequently, the heart rate was not decreased immediately, but decreased gradually 1 h after lunch. A significant decrease of the LF/HF (high vagal drive) was evident 1 h after lunch. We suggest that this is the cause of the gap that appeared in the high heart rate and low LF/HF ratio after lunch.

We also found a strongly negative correlation between heart rate and LF and HF, and a weakly positive correlation between heart rate and the LF/HF ratio; these tendencies were found to be the same before and after a meal (data not shown). However, after a meal, there was a greater change in heart rate variability. Thus, meal intake has a greater effect on LF, HF, and LF/HF ratio than fasting. One hour after a meal heart rate begins to decrease and the HF component increases. Therefore, we consider that the time point (1 h after a meal) whereby heart rate decreases is the point to better understand the autonomic nerve reaction. We suggest that the decreased LF/HF ratio shows that autonomic nervous balance is related to digestion as well as the correlation with the heart rate. We also consider that the point of decreased LF/HF ratio may reflect parasympathetic nervous system dominance by digestion after a meal.

Although we also investigated the relationship between heart rate variability and BMI in all subjects, we found no correlations between most of the individual components and BMI. Only the LF component 1 h after lunch appeared to be weakly correlated with BMI. In previous studies, the relationships between heart rate variability and BMI in healthy humans have also been unclear (Molfino et al. 2009; Sztajzel et al. 2009; Yi et al. 2013). We considered that the relationships between the ANS and BMI in healthy humans would not be clearly explained using a simple study design. Therefore, we chose a design that combined ANS responses after a meal and the influence of outdoor temperature (an interaction design). We undertook additional analysis of the means of all heart rate variability components at all time points between the two BMI groups (data not shown). We found no significant differences; this agrees with the findings of another similar study in which the mean total power (TP) and VLF/TP ratio were compared in two groups of differing BMI (Matsumoto et al. 2001).

We found a larger fall in the VLF component after lunch in the high BMI group. The significance of the VLF component is less clear, but it may reflect thermoregulatory vasomotor control exerted by the sympathetic nervous system (Kitney 1975; Akselrod et al. 1981; Thayer et al. 1997). Matsumoto et al. (2001) found that the strength of VLF component after a meal differed between the obesity and control groups. We also found a difference in the changes to the VLF component after a meal between the high and low BMI groups. We consider that the response of the VLF component after a meal may change if BMI is higher, although the VLF component generally decreases after a meal in human subjects. The VLF component, which suddenly decreases after a meal in the high BMI group, may lead to obesity. Further investigation is warranted to clearly understand the reaction of the VFL component reaction after a meal.

It is thought that the VLF component in obese women is lower than lean women (Matsumoto et al. 2001). Our findings suggest that increased BMI affects thermoregulatory vasomotor control after eating. We also found a significant change in the LF/HF ratio after lunch in the low BMI group, but not in the high BMI group. During digestion, the LF/HF ratio gradually decreases as the parasympathetic nervous system becomes dominant (Dionne et al. 2002; Harthoorn and Dransfield 2008). Therefore, our findings also suggest that an increased BMI affects the dominance of the parasympathetic nervous system after eating.

We also evaluated the ANS response to outdoor air temperature between the groups after eating lunch. We found significant relationships between the LF/HF ratio before and after lunch and outdoor temperature in the low BMI group. In particular, the LF/HF ratio before and 1 h after lunch was positively correlated with outdoor temperature in the low BMI group. This correlation was lost when BMI increased. It is thought that cold exposure increases sympathetic nerve activity in humans (Matsumoto et al. 1999). Our findings were not a consequence of the ANS response to cold exposure; rather indicate the influence of outdoor temperature on the ANS when the room temperature was constant. When the outdoor temperature is lower under otherwise normal conditions, dominance of the parasympathetic nervous system may increase both before and after lunch. In our opinion, this is the normal physiological response to eating when outdoor temperatures are cold in healthy women. Some of the changes in the components of heart rate variability appeared to be influenced by outdoor temperature in the high BMI group, implying that changes in the autonomic response to eating may trigger the metabolic syndrome in otherwise healthy humans. Furthermore, it could be said that the ANS reaction to food intake and outdoor temperature is a “blunted response”. Recently, Sartor (2013) suggested that changes of the gastrointestinal environment in obesity may lead to blunted vagal afferent signaling and disrupt sympathoinhibitory mechanism(s) in cardiovascular regulation after a meal. Our findings with respect to the heart rate variability response after lunch may be related to this study. Furthermore, the “blunted response” may be related to gastric hormones and cardiovascular regulation after a meal. In particular, gastric leptin may exert acute sympathoinhibitory and cardiovascular effects via vagal transmission with respect to short-term cardiovascular regulation (Sartor and Verberne 2010). We did not observe any significant reduction in the LF/HF ratio (high vagal tone or sympathoinhibition) 1 h after lunch in the high BMI group. Thus, the “blunted response” of the heart rate variability observed in our study suggest that changes in gastrointestinal hormones and the autonomic nervous system after a meal are influenced by increased BMI.

Many investigators have proposed that metabolic syndrome causes decreased sympathetic nerve activity and dysfunction or imbalance of the ANS (Matsumoto et al. 1999,2001; Tentolouris et al. 2003; Kataoka et al. 2004; Franklin et al. 2008; Boer-Martins et al. 2011; Sjoberg et al. 2011; Pal et al. 2012; Wijngaarden et al. 2012). We found clear changes in the autonomic response to eating and outdoor temperature in healthy women with high BMIs, which is an important contribution to the literature concerning metabolic syndrome.

Our study has several limitations. First, all subjects were young women and lived in Hiroshima, Japan, which may limit the generalizability of our findings. Notwithstanding, there is a large body of literature that has exclusively studied women. Second, we did not measure the distribution of adipose tissue. Some investigators have proposed that central adiposity is an important factor (Soares-Miranda et al. 2011; Andrew et al. 2013); however, as it is less common in women we chose BMI to define our groups (Stevens and Nowicki 2003). Our group was representative of healthy Japanese women. The mean BMI of Japanese women of similar age is 20.39 kg/m^2^, and in non-smoking Japanese women the BMI range with lowest all-cause mortality is 19.0–20.9 kg/m^2^ (Stevens and Nowicki 2003; Takimoto et al. 2004). Understanding the influence of BMI on the autonomic responses to eating may also explain some of the ambiguities of the metabolic syndrome, such as the obesity paradox: although many questions remain, we have shown that an increased BMI can affect the autonomic response to eating even in healthy people (Kaneko et al. 2013; Lavie et al. 2013).

The MONA LISA hypothesis proposed that diminished sympathetic nervous system activity was evident in most types of obesity (Bray 1991). The changes in the autonomic response to eating in obese people may be a consequence of their increased BMI. The differences in autonomic response that we found may have been differences in sensitivity rather than magnitude. The autonomic responses to food intake and environmental conditions are very important for homeostasis in humans. In particular, we think that increased BMI strongly affects the dominance of the parasympathetic nervous system after eating, and that environmental conditions may also affect appetite and metabolism. We speculate that long-term changes in these responses may help to establish personal homeostasis in healthy people.

## Conclusions

We found that some autonomic responses to eating appeared to be impaired by high BMI in healthy women. This finding may give clues as to the pathophysiological mechanisms that give rise to metabolic syndrome and obesity in otherwise healthy people, and may also explain the mechanisms underpinning the MONA LISA hypothesis. We propose that differences in autonomic responses to eating may trigger changes in human homeostasis.

## Methods

### Subjects

Fifty-five healthy Japanese female university students participated in this study, which was conducted between March 2010 and April 2012 (the subjects were only studied for one day). The study was approved by the Human Studies Committee of Hiroshima Bunka Gakuen Two-Year College. All subjects gave their informed consent. All were non-smokers, were not taking prescription medication, and had no history of cardiovascular or endocrine disease. Exclusion criteria were excessive weight loss during the previous 3 months and menstruation during the study. Participants were divided into a high BMI group (26 subjects; BMI >20.4) and a low BMI group (29 subjects; BMI <20.3) using a median split. On the day of participation, we recorded what the subjects ate for breakfast and confirmed that they had not ingested alcohol, caffeine, or capsaicin, had fasted since, and had slept well. Each subject underwent heart rate variability measurements on one day only (a holiday or nonworking day). Weight and height were measured while the subjects wore light indoor clothing with empty pockets and no shoes. BMI was calculated as weight divided by height squared. The proportion of body fat was measured using a BC-520 body composition meter (Tanita Corporation, Tokyo, Japan).

### Lunch and heart rate variability measurements

Heart rate variability is the physiological phenomenon of variation in the beat-to-beat interval, and is influenced by the parasympathetic and sympathetic nervous systems at the sinoatrial node. Spectral analysis of heart rate variability is a noninvasive means of evaluating cardiac autonomic nervous system (ANS) activity. Spectral analysis of heart rate variability comprises high frequency (HF) and LF components, which predominantly represent parasympathetic and sympathetic nerve activities, respectively. However, some recent studies have shown that the LF component is largely determined by the parasympathetic system (Rahman et al. 2011; Reyes del Paso et al. 2013).

The LF/HF ratio represents the balance between the two autonomic systems; an increase in the ratio suggests that the sympathetic nervous system is dominant (Akselrod et al. 1981; Pagani et al. 1986). Furthermore, the VLF component may reflect the influence of the thermoregulatory vasomotor control system on the sympathetic nervous system (Kitney 1975; Akselrod et al. 1981; Thayer et al. 1997).

Heart rate variability was calculated from individual datasets recorded using an SA-3000P device (Tokyo Iken Corporation, Tokyo, Japan). Each recording was 5 minutes long. Heart rate variability was calculated from the R-R intervals using power spectral analysis. Spectrum estimation was analyzed by fast Fourier transformation. Heart rate variability analysis was carried out based on the guidelines of the Task Force of the European Society of Cardiology and the North American Society of Pacing and Electrophysiology (1996). Spectral analysis was split into HF (0.15–0.40 Hz), LF (0.04–0.15 Hz), and VLF (0.0033–0.04 Hz) components. We calculated the LF/HF ratio, which reflects the balance between sympathetic and parasympathetic nervous system activities (Kitney 1975; Akselrod et al. 1981; Pagani et al. 1986; Thayer et al. 1997).

All heart rate variability measurements were taken at least 3 h after breakfast with the subjects wearing light indoor clothes. Subjects were asked to refrain from eating or performing any physical activity during the morning before the study. The subjects were provided with an identical lunch at noon (lunch was a Japanese meal, *gyudon*, consisting of rice and beef; total energy: 806 kcal, 66.4% carbohydrates, 12.8% protein, and 20.8% fat). The mean time taken to eat lunch was 16 min. The lunch contained no caffeine or capsaicin. Heart rate variability was measured before lunch, immediately after lunch, 30 min after lunch, and 1 h after lunch. The subjects were alone in a quiet, well-ventilated and lit room where the ambient temperature was maintained at 20–25°C; all measurements were taken after the participants had adapted to the room temperature for 1 h. (We did not consider it necessary to take outdoor measurements as people typically eat indoors).

### Outdoor environmental factors

Outdoor temperature, atmospheric pressure, and relative humidity data were obtained from the Hiroshima Local Meteorological Observatory. This institution is approximately 3 km away from the study location. The outdoor environmental data used were the mean of the values obtained from 11:30 am to 1:30 pm on the day that the measurements were taken.

### Data analysis

All statistical analyses were performed using Statistical Package for the Social Sciences software (SPSS for Windows, version 17.0; IBM SPSS, Tokyo, Japan) and Interaction! software (http://www.danielsoper.com/Interaction/). Descriptive statistics of all subjects and outdoor environmental factors are expressed as mean ± standard error (SE). Unpaired t-tests were used to compare the characteristics and outdoor environmental factors of the high and low BMI groups. The Wilcoxon signed-rank test was used to compare heart rate variability before and after lunch. In a graph, the Friedman test was performed to assess temporal changes in heart rate variability before and after lunch. Spearman’s rank correlation coefficients were used to express the extent of correlations between heart rate variability, BMI, and outdoor temperature. Multiple regression analysis was used to characterize the relationships between heart rate variability, BMI, and outdoor temperature. A simple slope test that showed the interaction of subjects’ BMI mean, +1 SD, and –1 SD was used to identify the relationship between the LF/HF ratio and outdoor temperature.

## Abbreviations

ANS: Autonomic nervous system
BMI: Body mass index
HF: High-frequency
LF: Low-frequency
VLF: Very-low-frequency
LF/HF: Low-to-high frequency ratio
TP: Total power.
